# Editorial: 2D nanostructures for electrocatalysis and electrochemical sensing

**DOI:** 10.3389/fchem.2023.1263648

**Published:** 2023-08-02

**Authors:** Shahid Zaman, Min Wang, Muhammad Asif, Leiming Hu

**Affiliations:** ^1^ Institut d’Innovations en Écomatériaux, Écoproduits et Écoénergies, Université du Québec à Trois-Rivières, Trois-Rivières, QC, Canada; ^2^ College of New Energy, China University of Petroleum (East China), Qingdao, China; ^3^ School of Chemistry and Chemical Engineering, Shanxi University, Taiyuan, China; ^4^ Department of Mechanical Engineering, Carnegie Mellon University, Pittsburgh, PA, United States

**Keywords:** two-dimensional materials, nanocomposites, renewable energy technologies, electrocatalysis, electrochemical sensors

Two-dimensional (2D) materials have emerged as a transformative class of materials, showing great promise in various fields, including electrocatalysis, photocatalysis and electrochemical sensing, thanks to their unique physical and chemical properties (Wang et al., 2023). The journey of 2D materials started with the discovery of graphene, a single layer of carbon atoms with remarkable electrical conductivity and high chemical stability. These materials showed excellent electrocatalytic activity for the hydrogen evolution reaction, and due to their suitable bandgap, they found use in photocatalytic reactions like water splitting (Zaman et al., 2022b). More recently, MXenes have been explored, demonstrating promising electrocatalytic activity for various reactions, including the hydrogen evolution reaction, the oxygen evolution reaction, and the nitrogen reduction reaction (Zhou et al., 2021).

Current research mainly focuses on enhancing the catalytic activity of 2D materials by engineering defects, doping with other elements, or altering the morphology. Similarly, designing hybrid structures by combining different 2D materials or integrating 2D materials with other nanostructures to boost the overall catalytic performance (Douka et al., 2020). Additionally, various lesser-known 2D materials, like boron nitride and black phosphorus, have been explored, revealing unique properties and applications. Although 2D materials have been versatile, the field must tackle the challenges of scaling lab-scale technologies to commercial production levels sustainably and economically (Zaman et al., 2022a). Despite these challenges, the future of 2D materials in catalysis remains bright, with myriad opportunities for revolutionizing energy systems and environmental remediation technologies.

Herein, we collected eight valuable contributions focusing on the synthesis and applications of 2D materials for electro and photocatalytic reactions. Among the eight contributions to this Special Research Topic, one review discusses the 2D materials for various electrocatalytic applications and seven original research focusing on 2D materials. Electrodeposition of Pt-Ni nanoparticles on graphene as an electrocatalyst for oxygen reduction reaction has been presented by Li et al., showcasing the 2D graphene role in ORR. Similarly, Sharif et al. successfully developed tuberculosis (TB) detection methods using Au-electroplated screen-printed electrodes as electro-DNA (E-DNA) sensors. The E-DNA sensors showed high sensitivity and specificity in differentiating TB-positive and TB-negative raw sputum samples, offering a rapid and cost-effective alternative for TB diagnosis.

Furthermore, Butt et al. synthesized nickel-doped lanthanum to create nanomaterials and evaluated their electrocatalytic properties for oxygen reduction reaction. The results demonstrated that the nanomaterials exhibited significantly enhanced ORR activity compared to pure lanthanum cerate. The presence of nickel dopants promoted the formation of active sites and facilitated the charge transfer process, leading to improved catalytic performance. Khan et al. discussed the applications of nanocomposites in electrochemical sensors and drug delivery systems in a review article. Nanocomposites have been widely utilized to enhance sensor platform sensitivity, selectivity, and stability, showing promising results in detecting various analytes, such as heavy metals and pollutants. In another article, Khan et al. outline the key findings of a lead-free ternary ceramic system that exhibits desirable characteristics for high energy storage applications. The fabricated ceramics demonstrate high dielectric constant, low dielectric loss, and excellent energy storage efficiency. Moreover, the materials exhibit good stability and reliability under various operating conditions.

Khan et al. developed green and sustainable lead oxide nanoparticles using a plant extract for photo-electrocatalytic degradation of organic pollutants. The green synthesis approach offers an eco-friendly alternative to conventional methods and enhances nanoparticle stability and performance. These findings suggest the potential application of lead oxide nanoparticles in environmental remediation and antimicrobial treatments with reduced environmental impact. Khan et al. developed cerium-based coatings to improve the electrochemical corrosion resistance of Al alloy 6101. This enhanced corrosion resistance is attributed to the cerium compounds’ ability to passivate the Al surface and inhibit the dissolution of the metal, thereby preventing degradation and increasing the alloy’s lifespan. Ren et al. studied the room-temperature ferromagnetic phase transformation in converting disordered oxygen vacancies to ordered oxygen chains. Their findings reveal that introducing Cu doping enhances the ferromagnetic properties and affects the crystal structure, inducing changes in lattice parameters and oxygen stoichiometry, resulting in increased saturation magnetization and improved magnetic ordering.

In our capacity as guest editors, we acknowledge the significant contributions made to this Research Topic and are appreciative of the reviewers’ constructive review. We anticipate that a larger readership will gain insight from this Research Topic regarding the 2D materials used in electrocatalysis.
